# Non-destructive imaging of buried electronic interfaces using a decelerated scanning electron beam

**DOI:** 10.1038/ncomms12701

**Published:** 2016-09-02

**Authors:** Atsufumi Hirohata, Yasuaki Yamamoto, Benedict A. Murphy, Andrew J. Vick

**Affiliations:** 1 Department of Electronics, University of York, Heslington, YO10 5DD York, UK; 2 SM Business Unit, JEOL, Musashino 3-1-2, Akishima, Tokyo 196-8558, Japan; 3 Department of Physics, University of York, Heslington, YO10 5DD York, UK

## Abstract

Recent progress in nanotechnology enables the production of atomically abrupt interfaces in multilayered junctions, allowing for an increase in the number of transistors in a processor. However, uniform electron transport has not yet been achieved across the entire interfacial area in junctions due to the existence of local defects, causing local heating and reduction in transport efficiency. To date, junction uniformity has been predominantly assessed by cross-sectional transmission electron microscopy, which requires slicing and milling processes that can potentially introduce additional damage and deformation. It is therefore essential to develop an alternative non-destructive method. Here we show a non-destructive technique using scanning electron microscopy to map buried junction properties. By controlling the electron-beam energy, we demonstrate the contrast imaging of local junction resistances at a controlled depth. This technique can be applied to any buried junctions, from conventional semiconductor and metal devices to organic devices.

## Abstract (web summary)

Imaging buried interfaces is necessary to assess the quality of electronic devices and their degradation mechanisms. Here, Hirohata *et al*. use energy-filtered scanning electron microscopy to image buried defects in an inorganic lateral spin-valve device, at the nanometre scale and non-destructively.

## Graphical abstract

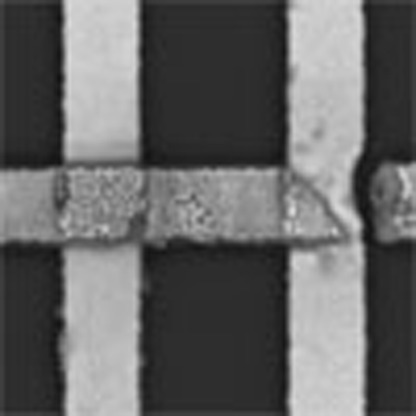

Almost all electronic (http://www.itrs.net/) and spintronic devices^1^ have junctions between different types of semiconductors (for example, p–n junctions) and metals and non-metals (for example, metal/semiconductor and tunnel junctions) at the core of their functionality. The efficiency and even the actual functionality of such devices are determined by the quality of the interfaces between the constituent layers. By definition, these interfaces in junction structures are buried beneath the surface layer and hence any defects, either in the form of pinholes or impurities, are impossible to be imaged without using time-consuming and complex techniques as summarized in Fig. 1. These techniques are based on microscopy, spectroscopy, scattering and reflection, and electrical methods. Microscopic techniques are the most powerful ones among them, such as cross-sectional transmission electron microscopy (TEM)^2^, and can reveal detailed information about atomic structures at the junction interfaces. However, the additional preparation required for a cross-sectional sample involves erosion and strain-induced damage of the junction and hence features that subsequently appear in the imaging process may be due to the sample fabrication process rather than being inherent in the original device. Helium ion microscopy (HIM) has also been used to observe the topology, while the helium ion beam can mill the sample *in situ*^3^. HIM can therefore achieve subsurface imaging on a semiconductor structure with sub-nm resolution but it is destructive to image buried junctions. To avoid such preparation processes, electron beam-induced current has been developed to image the distributions of the conductive properties of the buried junctions^4^. However, this technique is limited to conductive samples with the resolution of sub-micron and is difficult to make quantitative analysis on the junction properties, such as their conductivities and the position of the defects. For a semiconductor integrated circuit, voltage alteration techniques in scanning electron microscopy (SEM) can also be used but their resolution is typically in a micron scale^5^.

On the other hand, spectroscopic techniques can disclose the chemical composition in the vicinity of the junction interfaces and can be used in combination with other techniques, such as microscopic imaging. Secondary ion mass spectroscopy^6^, Auger electron spectroscopy^7^, energy dispersive X-ray spectroscopy and cathode luminescence^8^ have nanometric resolution but they require destructive sample preparation, typically Ar-ion bombardment, to expose the desired interfaces for analysis. By extending the penetration depth by employing different probing light or X-ray, attenuated total reflection-infrared spectroscopy^9^ or X-ray photoelectron spectroscopy) (http://srdata.nist.gov/xps/DataDefinition.aspx) can be used in a non-destructive way. However, these techniques superimpose all the information from the layers the beam penetrates through. These techniques have their resolution to be limited by their wavelength, that is, almost a sub-micron scale.

For junction evaluation techniques based on reflection and scattering, elipsometry has both nanometric resolution and non-destructiveness^10^. However, it requires an analytical model for fitting and is difficult to be correlated to transport properties. Rutherford backscattering^11^ and X-ray reflectivity^12^ have also been commonly used to evaluate the interfacial roughness microscopically, which again requires an analytical model for data fitting. To avoid such models, X-ray topography^13^ and grazing-incident small-angle X-ray scattering^14^ have recently been developed. These techniques still need more improvement, particularly in their resolution. In addition, a conventional current–voltage (*I–V*) measurement has been utilized to assess a macroscopic junction potential. For example, for a tunnel junction, Simmons' fitting^15^ is typically used to estimate the barrier height and thickness. Such electrical methods are important to determine the device performance but they cannot reveal microscopic transport properties.

In this study, a decelerated electron beam was used to control its penetration depth into a multilayered junction. The beam generated secondary and backscattered electrons after impacting on a junction layer under evaluation. By combining the control of the electron-beam voltage and energy filters, these electrons generated at a specified layer were collected to produce an image of conductance distributions across the buried junction. Our technique can be used as a quality assurance tool in nanoelectronic device fabrication.

## Results

### Electron-beam deceleration technique

**Figure 2:**
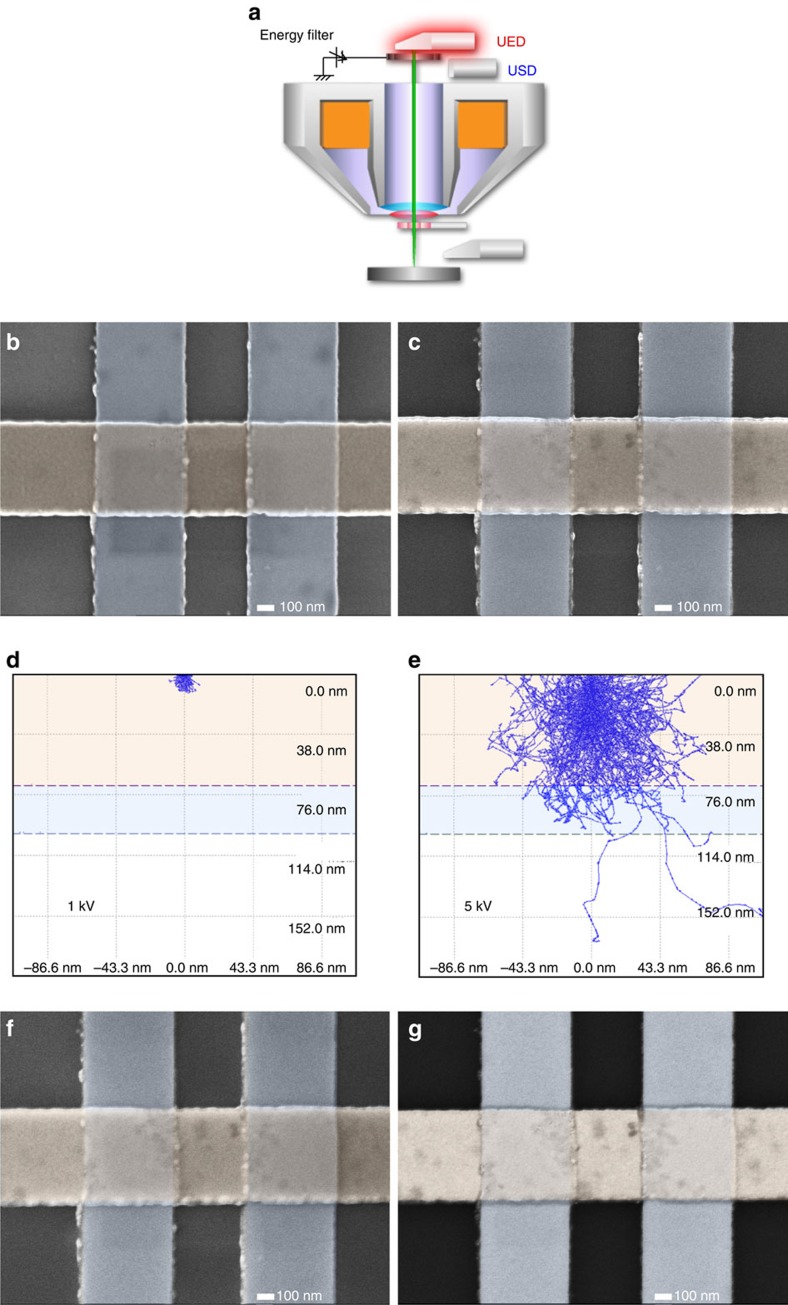
Imaging of the fresh lateral spin–valve with decelerated electron beam. (**a**) Proposed technique in this study for the junction evaluation by decelerating electron beam to control its penetration depth to meet the vertical position of the junction. The alignment of the upper electron detector (UED), the upper secondary electron detector (USD) and the energy filter used in this study is also shown. (**b**) Scanning electron microscopy (SEM) image taken by UED with the secondary electron (SE) mode at *V*_acc_=1 keV, which can penetrate into 10 nm below the surface. False blue and orange colour is provided for Ni_0.8_Fe_0.2_ and Cu wires. (**c**) Corresponding SEM image taken at *V*_acc_=5 keV, which can penetrate into 100 nm below the surface. (**d**,**e**) Interaction volume simulations to estimate the penetration depth for **b**,**c**. (**f**) SEM image taken by USD with the SE mode at *V*_acc_=5 keV using energy filter below −500 V as. (**g**) Corresponding SEM image taken by UED with the backscattered electron (BSE) mode at *V*_acc_=5 keV using energy filter above −500 V.

A field-emission scanning electron microscope (JEOL, JSM-7800F Prime) was used to develop a technique for potential mapping on buried junctions spanning a wide range of resistance. Here to maintain nanometric resolution, the initial electron beam was accelerated at (*V*_spec_+*V*_acc_) keV, where the bias voltage for specimen was −*V*_spec_ and the effective acceleration voltage landed on the specimen was *V*_acc_. This ensures high-imaging resolution, ∼0.7 nm resolution for *V*_spec_=5 keV, *V*_acc_=1 keV for example. In our observation, we managed to identify defects with their diameter of a few nanometres clearly (Fig. 2b,c). The beam was then decelerated in the vicinity of the specimen stage (the so-called gentle beam mode). This enabled us to control the beam penetration depth to match the vertical position of the buried junction interface under evaluation and the resulting secondary electrons (SE) and backscattered electrons (BSE) to be generated near the buried junction interfaces. In combination with an energy filter placed beneath the upper electron detector (UED) and the upper secondary electron detector (USD) (see Fig. 2a), more precise selection of the SE and BSE energies was achieved as detailed in Methods.

For SEM imaging, either SE, BSE or both has been commonly used. In the specimen, incident electrons are scattered inelastically and generate SE according to the energy loss during scattering events. Typically SE can be generated in the vicinity of the surface (approximately three times of the mean free path of SE, that is, around 3–10 nm depending on the materials). Emitted SE to a vacuum can then be detected to create a SEM image. At a decelerated electron-beam energy below a few 100 eV, the incident beam is easily scattered by the materials in the specimen, allowing the interactions between the beam and materials to occur within a few nanometres of the surface. The deceleration voltage between 500 eV and 5 keV has therefore been used in this study to avoid such scattering and to ensure the detection of electrons from the buried junctions.

In a junction, sharp and clean interfaces produce reproducible interfacial resistances. Such a junction may offer interfacial resistances ranging from a few pΩ m^2^ to μΩ m^2^ depending on the combinations at the junction interfaces, such as residual resists and oxides. For example, a spin–valve interface consisting of a ferromagnetic metal/non-magnetic metal/ferromagnetic metal multilayer typically shows an interfacial resistivity of over a few nΩ m^2^. However, defects, such as pinholes and impurities, show much smaller or larger resistivities, which can become of the order of a few pΩ m^2^ or larger than a few μΩ m^2^, respectively. These differences in resistivities generate a potential difference of a few eV up to a few keV between the different interfacial conditions. To detect such a potential difference, the electron beam needs to be decelerated effectively to the keV order and below. This deceleration voltage depends on the energy levels of SE and BSE from the buried interfaces to be observed. These energy levels can be simulated by using a well-established model (Monte Carlo Silulation of electron trajectory in solids, http://www.gel.usherbrooke.ca/casino/index.html).

### Fresh junction imaging

For a lateral spin–valve device consisting of two 200 × 200 nm Ni_0.8_Fe_0.2_/Cu junctions, SEM images were taken at two distinct effective accelerated voltages (*V*_acc_) of 1 and 5 keV as shown in Fig. 2b,c, respectively. Here the bottom 30-nm-thick Ni_0.8_Fe_0.2_ nanowires were fabricated using a combination of standard electron-beam lithography and a lift-off process-first, followed by top 70-nm-thick Cu wires (see Methods for more details). The device was then characterized by the conventional four-probe method as explained in Methods, confirming the resistance to be ∼5 nΩm^2^. As simulated in Fig. 2d,e, the effective voltage controls the depth profiles of the electron beam for imaging. This means the effective voltage of 1 keV allows the electrons to only penetrate into the surface Cu layer above the interface as shown in Fig. 2d, while that of 5 keV allows the electrons to penetrate through the Ni_0.8_Fe_0.2_/Cu junction (Fig. 2e). This clearly indicates that the effective electron-beam acceleration voltage in the vicinity of the specimen surface is one of the key parameters to determine the depth of the measurement positions for the buried junction imaging proposed in this study. This technique is much simpler than electric-field measurements using cross-sectional TEM imaging^16^ for example.

By comparing the two images in Fig. 2b,c, one can identify defects in the Cu nanowires, which can be seen as grey regions and black dots. The brightness in a SEM image created by SE is proportional to the number of generated SE in the specimen. The SE generation depends on the effects of surface morphology, specimen edges, acceleration voltages, atomic number of the specimen materials and charging-up on the specimen surface. Here the Cu and Ni_0.8_Fe_0.2_ wires are proven to have smooth surface without showing clear contrast from Fig. 2b. As seen in Fig. 2b,c, the edges of the device show bright contrast as expected. The two SEM images obtained at two different acceleration voltages did not show a clear difference in their general contrast. The device we observed was conductive and therefore should not induce any charging-up effect. We can hence exclude these effects and can conclude that the difference in the contrast indicates variance in the conductance across the specimen. The regions with darker contrast may therefore represent defects in the device, which generate fewer SE than the other majority regions. In Fig. 2b, some minor grey regions are seen in the Ni_0.8_Fe_0.2_ wires, indicating that the Ni_0.8_Fe_0.2_ wires have some defects near the surface. Figure 2b also shows that the junction regions do not have any contrast, confirming that the Cu wire has no defects near the surface. On the other hand, Fig. 2c shows some grey regions in the Cu wire, indicating that either within the Cu wire or the bottom interface of the Cu wire, that is, Cu/Ni_0.8_Fe_0.2_ and Cu/Si, has some defects. Such defects can also be seen at the junction regions. The size of the defects is measured to be between 10 and 100 nm. It should be noted that no grey regions are observed in the Ni_0.8_Fe_0.2_ wires, confirming the Ni_0.8_Fe_0.2_ wires have no defects at the Ni_0.8_Fe_0.2_/Si interface.

Another key parameter in this study is the energy filtering of SE and BSE from the specimen. For lateral spin-valve junctions, the voltage is applied at 1 and 5 keV, which controls the depth profile of imaging as discussed above. The energies of the generated SE and BSE then need to be selected to represent only the conductance difference in the buried junction. A similar study has been carried out on semiconductor p–n junctions^17^, confirming that the conductance difference induced by chemical potentials can be detected as SEM image contrast. To characterize the buried interfacial defects, more precise control of the electron energies to be detected for imaging is required, which can be carried out by an additional energy filtering at the detector and an additional decelerator attachment to the specimen stage and the control of the total layer thickness above the junctions^18^.

Figure 2f,g shows SEM images taken at *V*_acc_=5 keV using energy filters below −500 V with the SE mode and above −500 V with the BSE mode, respectively. Since SE can be emitted typically within 3 and 10 nm deep from the surface, the SE image shows almost identical contrast as that in Fig. 2c. In the BSE image in Fig. 2g, the defects in the Cu wire are unambiguously observed. In particular, almost a quarter of the junction is found to be covered by defects, such as at the top-right and bottom-left corners in the left hand side junction, and the left edge in the right hand side one. These defects suggest the presence of the contaminations at the junction interfaces, since they are typically non-conductive and do not produce SE and BSE. By comparing Fig. 2b,f,g, the interfacial defects are only found at the bottom Cu interface. These results prove that BSE have high spatial resolution and are highly sensitive to such defects in a buried junction.

**Figure 3:**
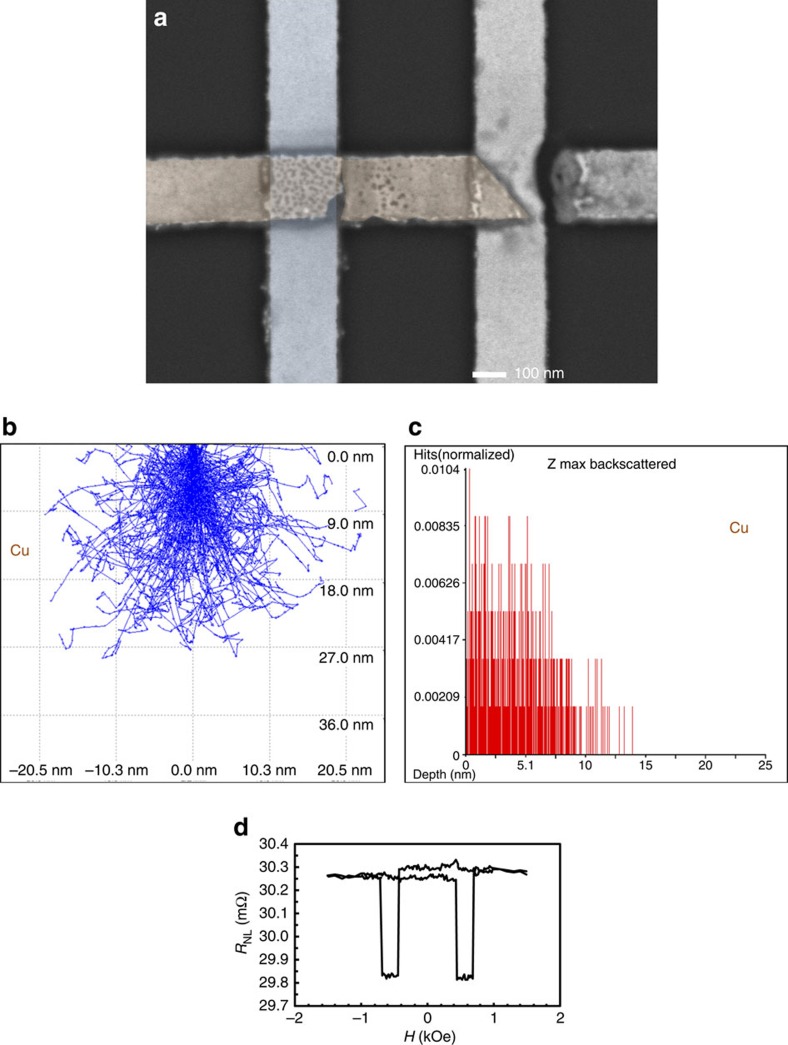
Imaging of the broken lateral spin–valve with decelerated electron beam. (**a**) Scanning electron microscopy (SEM) image of broken Ni_0.8_Fe_0.2_/Cu junctions taken by upper electron detector (UED) with the BSE mode at *V*_acc_=2.5 keV using energy filter above −500 V. (**b**) Interaction volume simulations to estimate the penetration depth for **a**. (**c**) Simulation of the generated backscattered electrons from the Cu layer. (**d**) Magnetoresistance result of the Ni_0.8_Fe_0.2_/Cu junction before the breakdown.

The giant magnetoresistance (GMR) behaviour of the device is shown in Fig. 3d, which confirms that spin-polarized electrons are successfully injected into the Cu wire and are efficiently detected by the Ni_0.8_Fe_0.2_ wire. This is measured under the non-local geometry at a current of 100 μA with showing the magnetoresistance ratio of 1.7% at room temperature as shown in Fig. 3d. This is similar to the values reported in similar devices^19^, assuring the quality of the lateral spin–valve junctions. The junction has then been damaged during the following measurements, showing the resistance to be increased up to a few μΩ m^2^.

### Broken junction imaging

After the junction breakdown, we observed the buried Ni_0.8_Fe_0.2_/Cu junctions as shown in Fig. 3a. Here *V*_acc_ of 2.5 keV is used, resulting the penetration of the electron beam to be down to ∼25 nm below the Cu surface (Fig. 3b). The penetrated electrons generate BSE in the Cu wire within ∼10 nm from the surface as simulated in Fig. 3c. Figure 3a reveals that many defects are formed in the broken Cu/Ni_0.8_Fe_0.2_ junctions and a part of the Cu wire is pealed off at the edges of the junctions. This suggests that the top Cu wire may be detached from the bottom Ni_0.8_Fe_0.2_ wire after junction breakdown, which is supported by the transport measurement with showing μΩ m^2^ resistivity. The black dots observed in the junctions are defects, which may be formed by detached interfaces. Such detached interfaces contain voids, which cannot generate BSE and can be shown as dark contrast in a SEM image. Similar defects are also seen in a part of Cu wire. These regions with many defects may be the area that can be detached by further current applications as seen in the right hand side junction in Fig. 3a. In summary, we can conclude that the deceleration technique has the capability to reveal defects and damage within the buried junctions in combination with standard simulations on electron scattering. Such a simple and non-destructive technique can be applied for other junctions by combining and controlling the deceleration voltage and energy filtering. This technique is expected to offer quality assurance for a wide range of electronic devices, consisting of nanoscale junctions (see GaAs/Fe and Fe/MgO/Fe junctions in Supplementary Figs 1 and 2, respectively). As discussed in the Supplementary Discussion, these junctions also show some contrast in their SEM images with a decelerated beam, proving the validity of the non-destructive method as shown above. This may allow further stacking and miniaturization of junctions to sustain the advancement in their density and functionality.

## Methods

### Device fabrication

The lateral spin–valve devices were fabricated by conventional electron-beam lithography and lift-off processes on a Si substrate with a thermally oxidised layer on the surface^20^. Two Ni_0.8_Fe_0.2_ nanowires were designed to be 30 nm thick and 200 nm wide with different shapes at their ends (square and sharp) to induce a difference in their magnetization-reversal fields. They were patterned using electron-beam lithography (JEOL, JBX-6300FS) and were deposited using high vacuum (HV) sputtering (SP) system (Leybold, UNIVEX 350). After their lift-off, these wires were bridged by a Cu nanowire (70 nm thick and 200 nm wide) made by the same manner. Before the Cu deposition, the surface of the Ni_0.8_Fe_0.2_ wires were cleaned by Ar-ion milling for 10 s at 50 W to remove oxides and contamination. Electrical contacts to these wires were finally made by photolithography (EGV, Mask Aligner) and lift-off process after the deposition of Cr (10 nm)/Au (150 nm) layers using an e-beam evaporator (Leybold, UNIVEX 350).

### Magnetotransport measurement

The transport properties of the lateral spin–valves were assessed by non-local magnetoresistance measurements with a dc reversal method^21^. Here an electrical current of 100 μA and an external magnetic field of up to ±1.2 kOe along the Ni_0.8_Fe_0.2_ nanowires were used for the measurements at room temperature.

### SEM observation

In the JSM-7800F Prime SEM, two detectors, an UED and an USD, are located above the lens and two additional detectors, a retractable backscattered electron detector and a lower electron detector, are installed just above the sample space (http://www.jeol.co.jp/en/products/detail/JSM-7800FPRIME.html). The UED becomes sensitive to reflected electrons from the specimen with energies above 10 eV when a bias voltage (*V*) is applied to the energy filter beneath, while the USD senses those below 1 eV. These detection modes with energy deceleration in the range between +500 V and −2 keV can be achieved coincidently. In addition, the acceleration voltage for the electron beam can be controlled between 10 and 30 keV. Based on such controllability, 0.7-nm resolution is guaranteed at the effective electron-beam landing voltages of both 1 and 5 keV at the specimen by the manufacturer.

### Data availability

The data that support the findings of this study are available from the corresponding author upon request.

## Additional information

**How to cite this article:** Hirohata, A. *et al*. Non-destructive imaging of buried electronic interfaces using a decelerated scanning electron beam. *Nat. Commun.* 7:12701 doi: 10.1038/ncomms12701 (2016).

## Supplementary Material

Supplementary Information Supplementary Figures 1-2, Supplementary Discussion and Supplementary References

## Figures and Tables

**Figure 1:**
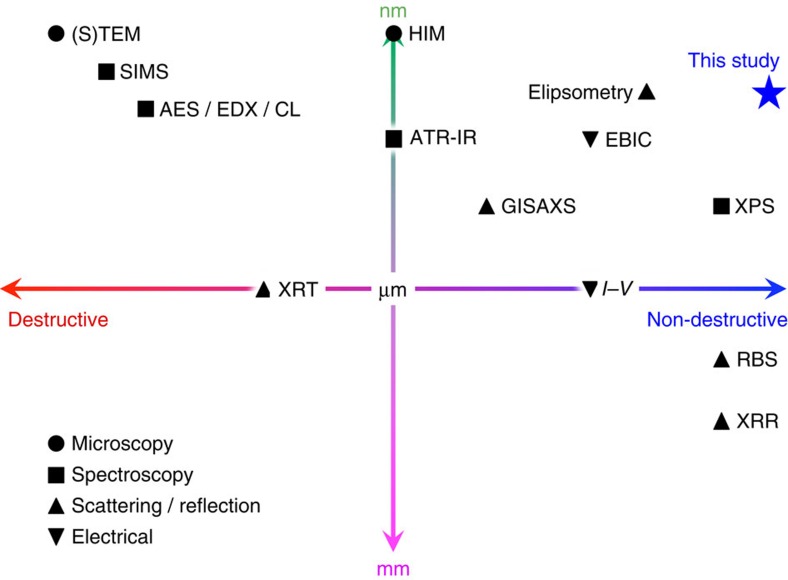
Evaluation techniques for embedded junctions. Major techniques for the evaluation of buried junctions against destructiveness and resolution, including those based on microscopy (closed circles), spectroscopy (closed squares), scattering and reflection (closed triangles), and electrical methods (closed inverse triangles). For the microscopic methods, (S)TEM and HIM denote (scanning) transmission electron microscopy and Helium ion microscopy. For the spectroscopic methods, SIMS, AES, EDX, CL, ATR-IR and XPS denote secondary ion mass spectroscopy, Auger electron spectroscopy, energy dispersive X-ray spectroscopy, cathode luminescence, attenuated total reflection-infrared spectroscopy and X-ray photoelectron spectroscopy, respectively. For the scattering and reflection methods, XRT, GISAXS, RBS and XRR represent X-ray topography, grazing-incident small-angle X-ray scattering, Rutherford backscattering and X-ray reflectivity, respectively. For the electrical methods, *I–V* indicates current–voltage measurements.
