# Supplementary material for: Non-destructive imaging of buried electronic interfaces using a decelerated scanning electron beam
Source: Nat Commun. 2016 Sep 2;7:12701. doi: 10.1038/ncomms12701 (PMC5025776; doi:10.1038/ncomms12701)
Supplement: Supplementary Information — Supplementary Figures 1-2, Supplementary Discussion and Supplementary References [file ncomms12701-s1.pdf]

Supplementary information

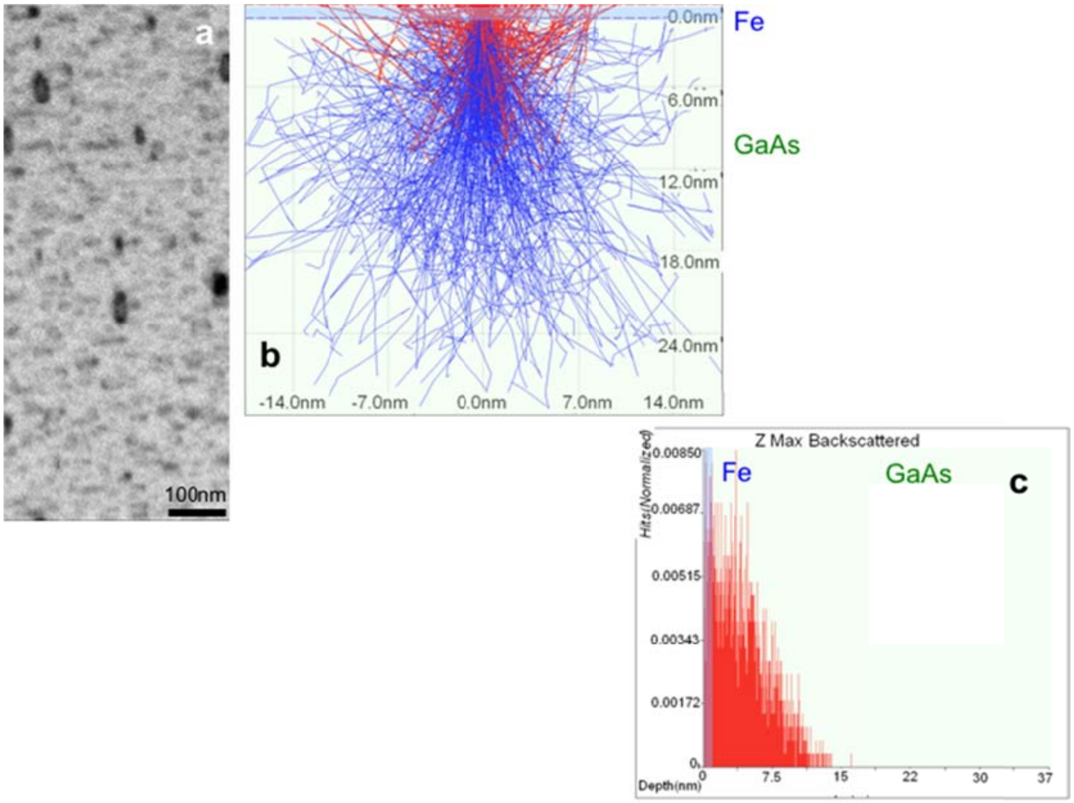

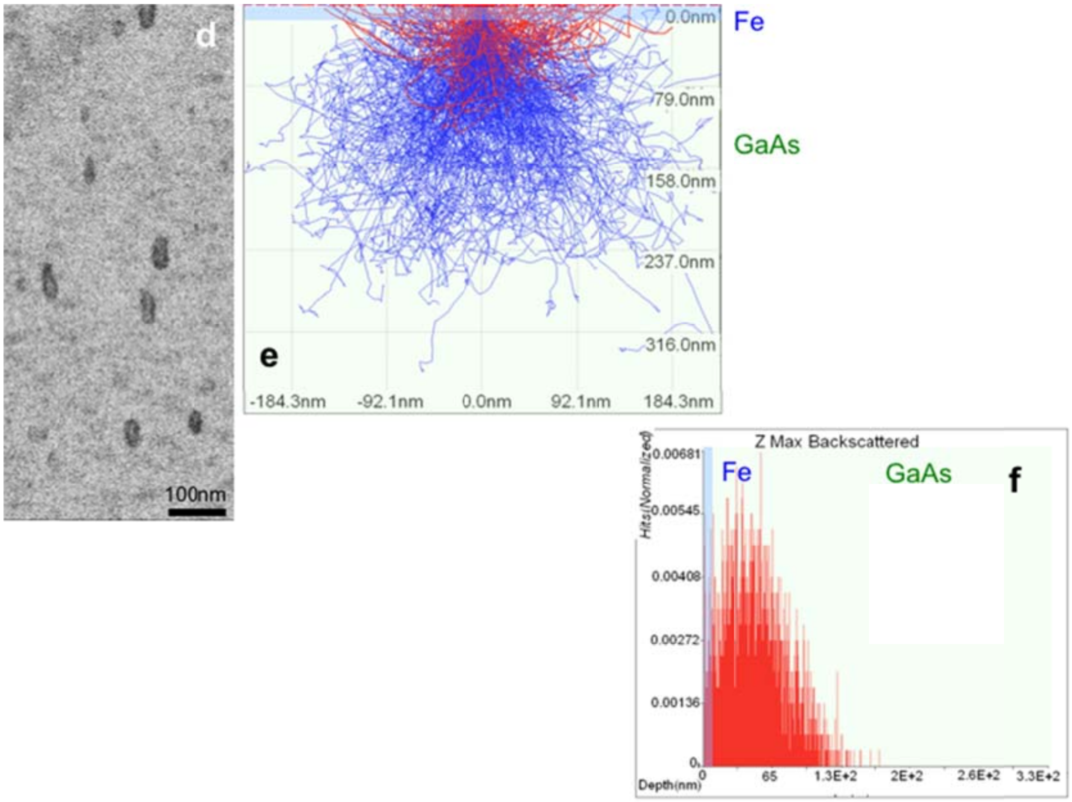

Supplementary Figure 1 Imaging of the Schottky juncstion with decelerated electron-beam. a, SEM image taken at V_{acc} = 1.5 keV. **b,** Interaction volume simulations to estimate the penetration depth for the backscattered electrons (red lines) and incident electron-beam (blue lines). **c,** Simulation of the generated backscattered electrons from the Fe and GaAs layers. **d,** Corresponding SEM image taken at V_{acc} = 7.0 keV. **b,** Interaction volume simulations to estimate the penetration depth for the backscattered electrons (red lines) and incident electron-beam (blue lines). **c,** Simulation of the generated backscattered electrons from the Fe and GaAs layers.

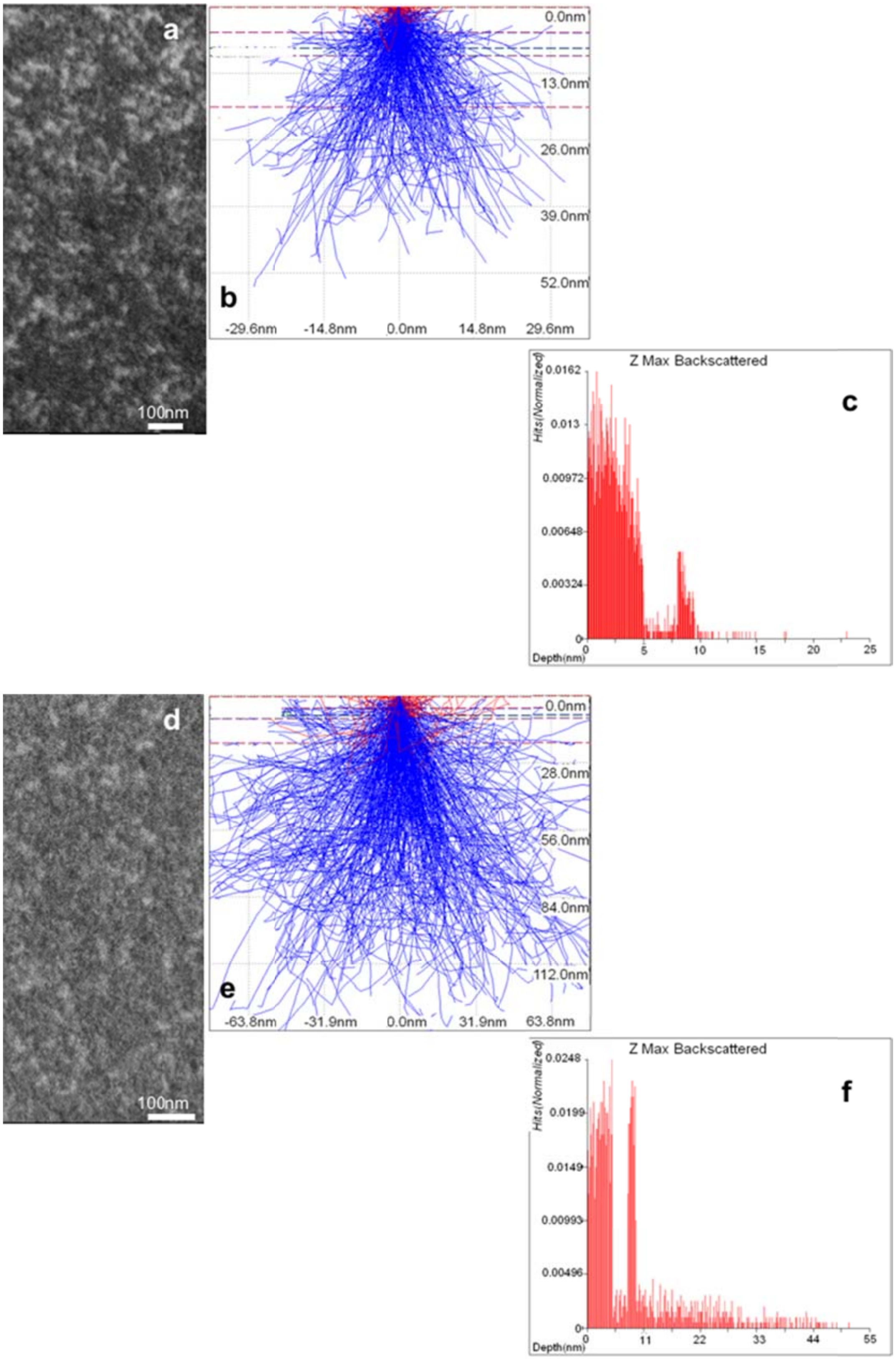
a
100nm
0.0nm
13.0nm
26.0nm
39.0nm
52.0nm
b
-29.6nm
-14.8nm
0.0nm
14.8nm
29.6nm
Z Max Backscattered
c
Hits(Normalized)
0.0162
0.013
0.00972
0.00648
0.00324
0
Depth(nm)
0
5
10
15
20
25
d
100nm
0.0nm
28.0nm
56.0nm
84.0nm
112.0nm
e
-63.8nm
-31.9nm
0.0nm
31.9nm
63.8nm
Z Max Backscattered
f
Hits(Normalized)
0.0248
0.0199
0.0149
0.00993
0.00496
0
Depth(nm)
0
11
22
33
44
55

Supplementary Figure 2 Imaging of the magnetic tunnel junction with decelerated electron-beam. a, SEM image taken by UED with the BSE mode at V_{acc} = 1.5 keV using energy filter above -500 V. **b,** Interaction volume simulations to estimate the penetration depth for the backscattered electrons (red lines) and incident electron-beam (blue lines). **c,** Simulation of the generated backscattered electrons from the two Fe layers. **d,** Corresponding SEM image taken by UED with the BSE mode at V_{acc} = 2.5 keV using energy filter above -500 V. **b,** Interaction volume simulations to estimate the penetration depth for the backscattered electrons (red lines) and incident electron-beam (blue lines). **c,** Simulation of the generated backscattered electrons from the two Fe layers.

Supplementary Discussion

Evaluation of metal/semiconductor junctions. A 1 nm thick epitaxial Fe film was deposited on a GaAs(001) substrate using ultrahigh vacuum (UHV) molecular beam epitaxy (MBE). We employed our recently optimised growth process to achieve an abrupt GaAs(001)/Fe interface[1]. The film was evaluated by the above deceleration technique. As shown in Suppl. Fig. 1a, a SEM image observed at V_{acc} = 1.5 keV shows some black spots with the size up to 500 nm and many grey dots with the size of approximately 20 nm. For this deceleration voltage, electron flight simulations confirm that the electron beam penetrates into the depth of 24 nm and the resulting BSE is predominantly generated from the Fe/GaAs interface as seen in Supplementary Figures 1b and 1c. The corresponding SEM image observed at V_{acc} = 7.0 keV, on the other hand, shows similar defects but grey dots become much weaker (see Suppl. Fig. 1d). Since the simulations indicate the electron beam penetrates into the depth of 250 nm and the resulting BSE is predominantly generated from the GaAs substrate as seen in Supplementary Figures 1e and 1f. By comparing these two images obtained at two distinctive deceleration voltages, this sample has two types of defects, black approximately 500 nm spots representing defects in GaAs and grey ~20 nm dots characterising Fe/GaAs interfacial defects. This again confirms that the proposed technique can detect the defects at the metal/semiconductor interfaces as well as those in bulk semiconductors.

Evaluation of magnetic tunnel junctions. An epitaxial magnetic tunnel junction, consisting of MgO buffer (10 nm)/Fe (5 nm)/MgO (1.5 nm)/Fe (3 nm)/MgO cap (3 nm), was deposited

on a MgO(001) substrate using ultrahigh vacuum (UHV) molecular beam epitaxy (MBE). The junction was evaluated by the above deceleration technique. As shown in Suppl. Fig. 2a, a SEM image observed at V_{acc} = 1.5 keV shows many grey dots with the size of 10~20 nm. For this deceleration voltage, electron flight simulations confirm that the electron beam penetrates into the depth of 30 nm and the resulting BSE is predominantly generated from the two Fe layers as seen in Suppl. Figs. 2b and 2c. The corresponding SEM image observed at V_{acc} = 2.5 keV, on the other hand, shows similar defects but grey dots become much weaker (see Suppl. Fig. 2d). Since the simulations indicate the electron beam penetrates into the depth of 120 nm and the resulting BSE is also generated from the two Fe layers as seen in Suppl. Figs. 2e and 2f. By comparing these two images obtained at two distinctive deceleration voltages, this sample may have some defects as appeared as grey dots in both images. However, since BSE can only be generated at the Fe layers, more precise energy control is required to differentiate the contrasts associated with the different Fe/MgO interfaces.

Supplementary references

1. Fleet, L. R., Yoshida, K., Kobayashi, H., Kaneko, Y., Matsuzaka, S., Ohno, Y., Ohno, H., Honda, S., Inoue, J. and Hirohata, A., Correlating the interface structure to spin injection in abrupt Fe/GaAs(001) films. *Phys. Rev. B* **87**, 024401 (2013).
